# Image-Based Deep Learning Detection of High-Grade B-Cell Lymphomas Directly from Hematoxylin and Eosin Images

**DOI:** 10.3390/cancers15215205

**Published:** 2023-10-29

**Authors:** Chava Perry, Orli Greenberg, Shira Haberman, Neta Herskovitz, Inbal Gazy, Assaf Avinoam, Nurit Paz-Yaacov, Dov Hershkovitz, Irit Avivi

**Affiliations:** 1 Hematology Division, Tel Aviv Sourasky Medical Center, Tel Aviv 6423906, Israel; 2 Sackler Faculty of Medicine, Tel Aviv University, Tel Aviv 6997801, Israel; 3 Pathology Department, Tel Aviv Sourasky Medical Center, Tel Aviv 6492601, Israel; 4 Imagene AI Ltd., Tel Aviv 6721409, Israelnurit@imagene-ai.com (N.P.-Y.)

**Keywords:** diffuse large B-cell lymphoma (DLBCL), high-grade B-cell lymphoma (HGBL), double hit, *MYC* rearrangement, *BCL2* rearrangement, artificial intelligence, deep learning

## Abstract

**Simple Summary:**

Double/triple-hit lymphomas (DHLs/THLs) are an aggressive type of high-grade B-cell lymphomas (HGBLs), characterized by translocations in *MYC* and *BCL2/BCL6*. DHL patients respond poorly to standard chemoimmunotherapy regimens; thus, timely and accurate diagnosis is of paramount importance for their proper clinical management. The standard technique used for the identification of these translocations is fluorescence in situ hybridization (FISH), which is not routinely performed at every medical center to all potential patients. In the current study, we employed an image-based, artificial intelligence, deep learning algorithmic tool for the identification of DHL/THL cases by analyzing scanned histopathological H&E-stained tissue slide images. Our preliminary results demonstrate high performances, suggesting the potential use of such a solution in the clinical workflow to support the management of HGBL patients.

**Abstract:**

Deep learning applications are emerging as promising new tools that can support the diagnosis and classification of different cancer types. While such solutions hold great potential for hematological malignancies, there have been limited studies describing the use of such applications in this field. The rapid diagnosis of double/triple-hit lymphomas (DHLs/THLs) involving *MYC*, *BCL2* and/or *BCL6* rearrangements is obligatory for optimal patient care. Here, we present a novel deep learning tool for diagnosing DHLs/THLs directly from scanned images of biopsy slides. A total of 57 biopsies, including 32 in a training set (including five DH lymphoma cases) and 25 in a validation set (including 10 DH/TH cases), were included. The DHL-classifier demonstrated a sensitivity of 100%, a specificity of 87% and an AUC of 0.95, with only two false positive cases, compared to FISH. The DHL-classifier showed a 92% predictive value as a screening tool for performing conventional FISH analysis, over-performing currently used criteria. The work presented here provides the proof of concept for the potential use of an AI tool for the identification of DH/TH events. However, more extensive follow-up studies are required to assess the robustness of this tool and achieve high performances in a diverse population.

## 1. Introduction

Diffuse large B-cell lymphoma (DLBCL) is an aggressive lymphoma. It is the most common type of non-Hodgkin’s lymphoma (NHL), accounting for approximately 30–40% of NHL cases. More than 20 years ago, gene expression profiling stratified DLBCL into three main subgroups, according to the cell of origin (COO); germinal center B-cell-like (GCB), activated B-cell-like (ABC) and unclassified (“type 3”) DLBCL [1,2]. These groups are characterized by different gene expression patterns, with a more favorable prognosis for GCB-type lymphoma compared to ABC.

An additional layer of B-cell lymphoma classification includes the presence of chromosomal rearrangements. MYC, a master regulator of multiple cellular processes such as cell proliferation, apoptosis and differentiation, is one of the most commonly rearranged oncogenes in B-cell lymphoma [3]. MYC deregulation can be found in many cancers, and translocations involving this oncogene occur in 5–15% of patients with DLBCL [4,5].

High-grade B-cell lymphoma (HGBL) was established as a distinct category of B-cell lymphomas in the 2016 revision of the World Health Organization (WHO) classification of lymphoid neoplasms [6]. A unique group among high-grade lymphomas, characterized by specific gene rearrangements, these lymphomas carry translocations in *MYC* together with one, or both, of the anti-apoptotic proto-oncogenes, *BCL2* and *BCL6*. HGBL, reported in less than 10% of cases of diffuse large B-cell lymphoma (DLBCL), have been referred to as double-hit (DH) or triple-hit (TH) lymphomas, if two or all three rearrangements are demonstrated, respectively.

Patients with HGBL-DH/TH poorly respond to R-CHOP (rituximab, cyclophosphamide, doxorubicin, vincristine and prednisone) chemoimmunotherapy and are at increased risk for central nervous system (CNS) involvement; therefore, other therapeutic regimens, aiming to overcome R-CHOP resistance and ensure CNS penetration, are often considered [7]. These include dose-adjusted R-EPOCH (rituximab, etoposide, prednisone, vincristine, cyclophosphamide, doxorubicin) [8], R-CODOX-M/IVAC (rituximab cyclophosphamide, vincristine, doxorubicin and high-dose methotrexate alternating with ifosfamide, etoposide and high-dose cytarabine) [9] and hyper-CVAD-R (cyclophosphamide, vincristine, adriamycin and dexamethasone with rituximab, alternating with methotrexate and cytosine arabinoside) [10,11].

The current diagnosis of DHL relies on fluorescence in situ hybridization (FISH) analysis, demonstrating *MYC* rearrangements together with *BCL2*//*BCL6* translocations [12]. However, using FISH is costly and requires elaborate laboratory protocols, which are not routinely performed at every medical center. Moreover, FISH results are usually not instantly attained, so the “DH molecular status” is often unknown by the time the first course of chemoimmunotherapy is administered. Thus, in most patients with DHL, the standard R-CHOP regimen is used at least in the first cycle of treatment and not a more intense protocol. Therefore, efforts are made to define restrictive criteria in order to identify the cases where FISH for DHL needs to be employed. The Ki-67 proliferation index (often high in patients with DHL), immunohistochemical (IHC) expression of c-MYC or diagnosis of the germinal GCB COO subtype have all been suggested [13], yet were found to be insufficiently specific and were associated with unacceptable rates of false negative cases [5].

The current diagnosis practices range from the performance of FISH analysis in all large B-cell lymphoma cases to a highly selective approach, restricting the analysis only to very suspicious clinical–pathological cases. While the first approach ensures accurate diagnosis, it is associated with considerable resources and high costs [14]. Conversely, the latter approach reduces efforts and costs, but increases the risk of missing DHL cases.

Deep learning (DL) applications are being extensively explored in digital pathology as novel solutions for cancer diagnosis [15,16]. The use of DL for both histopathology and molecular image-based analysis using the hematoxylin and eosin (H&E)-stained tissue slides can provide an immediate, objective and scalable solution that is exceedingly needed. This necessity is specifically prominent in hematopathology, where the microscopic diagnosis of hematological malignancies can be extremely challenging and molecular screening is not always available [17,18,19]. Indeed, there have been several publications in the last few years describing the potential use of DL for the identification and diagnosis of different subtypes of lymphoma, including DLBCL, using H&E-stained tissue slide images [20,21,22,23,24,25]. This approach of applying machine learning for the diagnosis of hematological neoplasms and DLBCL specifically is showing promising results and could significantly enhance the diagnostic process. However, the diagnosis and classification of hematological malignancies often rely not only on histopathological features but also on the characterization of genetic alterations [26].

Deep learning, which is adept in extracting relevant features from complex, variable data, is emerging as a powerful tool for identifying morphological patterns associated with molecular alterations from digitized histological slides [27]. Although the use of DL for the detection of a variety of molecular alterations directly from H&E slides is being widely investigated in diverse cancer types [28,29,30,31], its application for detecting genetic changes in lymphoma remains notably limited. A recent study, investigating the use of DL for the inference of *MYC* rearrangements from biopsies of patients with aggressive B-cell lymphoma, confirmed the potential value of this technology, demonstrating a sensitivity of 0.93 [32]. However, the specificity was only 0.52, attributing to high false positive rates of more than 30% [32]. Thus, despite their potential, DL solutions for the prediction of molecular alterations has not been broadly adopted in clinical settings yet, as they do not display high enough accuracies in robust large-scale studies [31].

Given the promising capabilities of DL and the importance of the identification of rearrangements for the proper management of DLBLC and HGBL patients, we chose to explore the abilities of AI tools in detecting DH/TH events in lymphoma specimen slides. In this study, we present a digital image-based approach, employing DL algorithms to differentiate between DHL/THL and non-DHL/THL cases by analyzing scanned images of H&E-stained tissue slides. While our work still requires further validation in a broader study, our DL classifier demonstrated high accuracies, suggesting that such an approach has the potential to be beneficial within hematological clinical settings.

## 2. Materials and Methods

### 2.1. Study Population

Patients diagnosed with aggressive B-cell lymphomas (DLBCL and HGL) between January 2017 and January 2022 at the Tel Aviv Sourasky Medical Center (TASMC), who were analyzed through FISH as part of their pathological workup, were included in this study. Patients with non-informative FISH results, attributed to technical issues, were excluded. The scanning of whole slide images (WSIs) of patients’ H&E-stained diagnostic slides was performed at 40× magnification, using a Philips Ultra-Fast Scanner (Philips Digital Pathology Solutions, Philips, Best, The Netherlands). Thirty-two biopsies from 30 patients were included in the training set, including 27 non-DH biopsies and 5 DH biopsies. The validation set included 25 cases, 15 non-DH and 10 DH/TH cases.

This study was approved by the Ethics Committee at the Tel Aviv Sourasky Medical Center (IRB 0308-22-TLV).

### 2.2. Data Collection

Data including patient demographics; clinical and laboratory characteristics at presentation (ECOG performance status, disease stage at diagnosis, lactic dehydrogenase (LDH) levels); the final results of histological diagnosis (DLBCL or HGBL); the results of immunohistochemistry staining focusing on BCL2, BCL6, c-MYC, CD10, MUM-1 and Ki-67; and the results of the FISH analysis for *MYC*, *BCL2* and *BCL6* were all collected from the patients’ electronic medical records.

### 2.3. Histopathological Analysis and Immunohistochemical (IHC) Staining

Immunohistochemical staining was performed using the following antibodies: anti-CD10 (clone 56C6, Master Diagnostica, Sevilla, Spain), anti-BCL6 (clone GI191E/A8, Cell Marque, Rocklin, CA, USA), anti-MUM-1 (clone MRQ-8, Cell Marque, Rocklin, CA, USA), anti-c-Myc (clone EP121, Cell Marque, Rocklin, CA, USA), anti-BCL2 (clone E17, Cell Marque, Rocklin, CA, USA) and anti-Ki-67 (clone SP6, Cell Marque, Rocklin, CA, USA). Staining was performed on the Ventana Ultra Benchmark (Ventana Medical Systems, Tucson, AZ, USA) automatic slide stainer. Positivity for the expression of CD10, BCL6 and MUM-1 was defined as 30% or more positive cells, 50% or more for BCL2 and 40% or more for c-MYC. The COO was determined using the HANS algorithm [33].

### 2.4. Fluorescence In Situ Hybridization (FISH) Analysis

FISH analysis was performed to assess *MYC* rearrangements using the Vysis MYC Break Apart FISH Probe Kit; *BCL2* rearrangements were assessed using the Vysis LSI IGH/BCL2 Dual Color, Dual Fusion Translocation Probe; and *BCL6* rearrangements were assessed using the Vysis LSI BCL6 (ABR) Dual Color Break Apart Rearrangement Probe (Abbott Molecular, Des Plains, IL, USA) and an automated fluorescence microscope scanning system (BioView Duet workstation; BioView Ltd., Rehovot, Israel). One hundred tumor cells at a minimum were evaluated per sample (except for rare cases where a minimum of 50 cells were evaluated). A cutoff of 10% was used to determine the positivity for each rearrangement.

### 2.5. Algorithm Development and Application

#### 2.5.1. Model Training

For the training of the DHL model, a self-supervised scheme with dynamic data augmentation, combined with multiple instance learning (MIL) algorithms, were applied.

In the self-supervised step, the model is pre-trained on large numbers of unlabeled histopathology slides. This initial step establishes a foundation model that can be adapted to various downstream tasks using limited numbers of training samples. As such, this approach is particularly useful in the field of histopathology and is increasingly being adopted, given the limited availability of labeled samples [34,35].

In the following fine-tuning step, in addition to the foundation model, multiple instance learning (MIL) is utilized and training is performed on labeled data. Each WSI is subdivided into multiple smaller patches which are used as input for modeling. Given that the labeling is assigned on a slide level and the model receives multiple patches that collectively represent the entire slide, the MIL approach, which allows one to make use of such weakly labeled data and provides a single classification for the entire slide, is a powerful technique for classifying WSIs [35,36].

**Figure 1:**
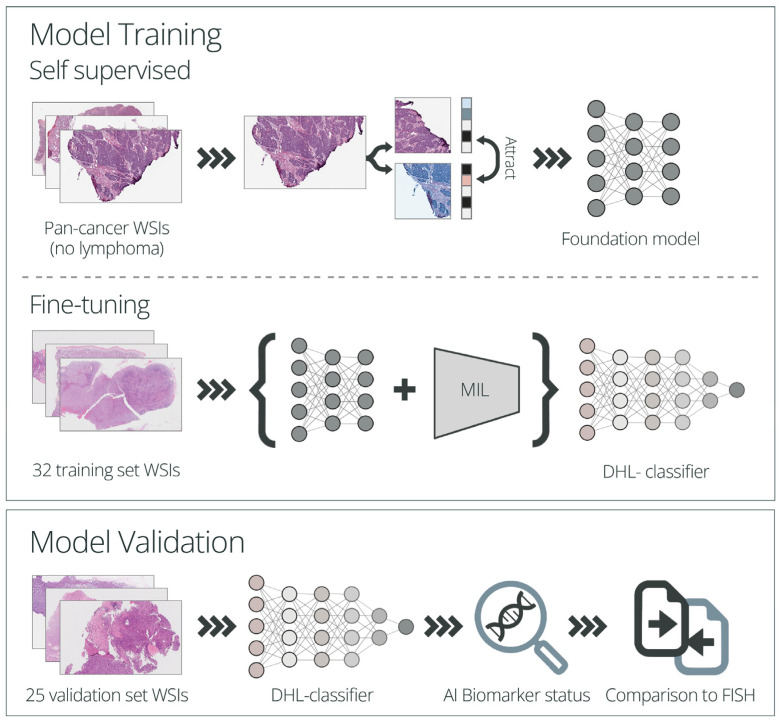
Study schematic representation. A self-supervised step was performed on a pan-cancer cohort (including cases of solid cancers with no lymphoma biopsies), establishing a foundation model, followed by a fine-tuning step using the training set’s WSIs, generating the final DHL-classifier. For the DHL-classifier performance evaluation, the DH/TH status of 25 cases included in the validation set was evaluated, and the results were compared to the official results reported in the FISH analysis. WSI—whole slide image, MIL—multiple instance learning, DHL—double-hit lymphoma, DH—double-hit, TH—triple-hit.

The self-supervised step was performed using untagged pan-cancer (not including lymphoma biopsies) WSIs of FFPE H&E-stained tissues scanned at 40× or 20× magnification from Imagene’s internal database (including slides from the TCGA research network). All 40× images were transformed to 20× for analysis. Data augmentation was performed using over 20 techniques, including color jitter and channel shuffle. For model fine-tuning and the generation of the final DHL, the foundation model, together with MIL, were applied on the lymphoma WSIs training set described above, using patches of 384 × 384 pixels. Training was performed for 20 epochs using a categorical cross-entropy loss, the Adam optimizer and a learning rate of 0.0001 (Figure 1).

#### 2.5.2. Model Performance Evaluation

For the validation step, a categorical prediction (positive/negative) was made using the DHL-classifier model (comprising both the foundation model and the MIL algorithm) and the results were compared to the FISH results for the *MYC* and *BCL2/6* rearrangements (Figure 1).

## 3. Results

### 3.1. Patient Characteristics

This study included 57 biopsies from 55 patients, divided into a training set, which included 32 biopsies, and a validation set, which included 25 unique patients. The characteristics of the patients are presented in Table 1. Biopsies from lymph nodes represented approximately a third of the samples (39%, *n* = 22), and the rest were biopsies from extranodal tissues (61%, *n* = 35). The DHL/THL patients (*n* = 15) were mostly diagnosed with the germinal center B-cell (GCB) COO subtype determined based on IHC (73.3%, *n* = 11), with a median Ki-67 of 88% (range 40–100%) and c-MYC and BCL2 expression in 85.7% (*n* = 12/14, one with no available data) and 64.3% (*n* = 9/14, one with no available data) of the cases, respectively.

### 3.2. Digital Imaging Analysis

The DHL-classifier was developed using H&E-stained slide images of 32 biopsies (as described in the methods section) (training set) that included five cases of DHL. The DHL-classifier was then blindly validated on an independent validation set, containing 25 DLBCL/HGL cases. The algorithm results were compared to the official clinical diagnosis that was based on the FISH analysis for the *MYC*, *BCL2* and *BCL6* rearrangements.

**Figure 2:**
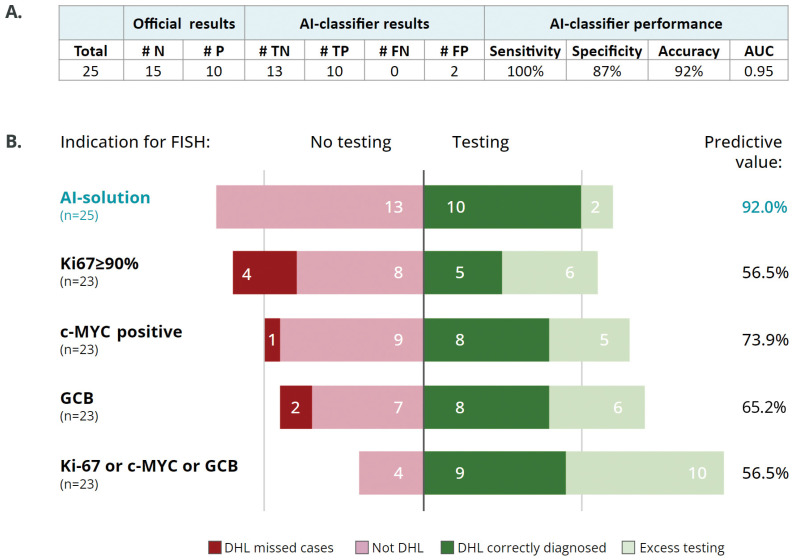
Performance of the DHL-classifier. (**A**) DHL-classifier results and performance in the validation cohort. N—negative, P—positive, TN—true negative, TP—true positive, FN—false negative, FP—false positive, AUC—area under the curve. (**B**) Predictive values of conventional methods vs. the DHL-classifier as a screening tool for FISH analysis. Presented are the number of samples in the relevant bars and predictive values for each screening method used. The number on the bars represents the number of cases in the relevant group.

The validation set comprised nine DHL cases, one THL case and fifteen non-DH DLBCL cases. Altogether, the model correctly identified all 10 DHL/THL cases, demonstrating 100% sensitivity. The specificity was 86.7% due to two false positive cases (negative for FISH analysis). The accuracy rate was 92% and the area under the curve (AUC) was 0.95 (Figure 2A).

### 3.3. AI Classifier as a Screening Tool for Selecting Cases for FISH Analysis

Current criteria for selecting cases for FISH analysis often rely on the presence of high Ki-67 (≥90%), the IHC expression of c-MYC and the classification of the GCB subtype. Therefore, we assessed the performance of these criteria, as well as the AI DHL-classifier, as a screening tool for referring cases for FISH analysis. The DHL classifier was found to provide a predictive value of 92%, compared with 57–74% for any of the three IHC evaluable criteria (Figure 2B and Table S1). The AI DHL-classifier displayed the highest sensitivity (100%) and specificity (87%) rates, with only two excess unnecessary tests and no missed DHL cases. The conventional screening criteria showed variable sensitivities, ranging from 56% to 89%, and variable specificities ranging from 54% to 64% (Table S1). When all three IHC parameters were used together as screening criteria (Ki-67 ≥ 90% or increased c-MYC expression or GCB subtype), the accuracy remained low (57%), with an excess of non-valuable tests; only four cases out of the entire evaluated cohort (*n* = 23) did not meet the FISH screening criteria.

## 4. Discussion

The outcome of DHL patients treated with R-CHOP is generally poor. Intensive treatment regimens such as dose-adjusted R-EPOCH, R-CODOX-M/IVAC and hyper-CVAD-R are often implemented in clinics to treat HGBL-DH/TH. Thus, the rapid and accurate diagnosis of DH/TH lymphoma is highly necessary. Unfortunately, diagnoses of DHL/THL are often delayed, or even missed, due to the limited availability of FISH tests, which are essential for establishing the diagnosis.

Here, we describe a deep learning-based algorithmic tool trained to detect DHL/THL, using H&E-stained biopsy slide images obtained from aggressive B-cell lymphoma patients. To evaluate the performance of our DHL-classifier, we used a cohort comprising images of samples that had been subjected to FISH during their diagnostic assessment and were not part of the training set. The cohort included, in total, 10 DHL/THL and 15 non-DH/TH samples. Our DHL-classifier identified all 10 DHL/THL cases, demonstrating 100% sensitivity. The specificity was 86.7%, with an AUC of 0.95 due to two false positive (FP) cases, where DH translocations were not identified through the FISH. Of note, cryptic rearrangements, undetected with FISH, may exist in up to 20% of DHL cases [37,38], raising the possibility that our FP cases might be due to “cryptic DHL changes”. However, this speculation requires further evaluation.

The rapid identification of patients with DHL, enabling the early upfront administration of more intensified and compatible therapies, is imperative. FISH analysis is the currently used method for the identification of patients that are positive for DH/TH-associated gene rearrangements. Testing all high-grade lymphoma cases, although ensuring the detection of most DHL/THL patients, is associated with increased diagnostic costs and a high testing burden. Given that several studies have shown that more than 20% of DHL/THL cases are of a non-GCB origin, 15–30% of *MYC*-rearranged cases fail to overexpress c-MYC, and at least a third of DHL cases exhibit Ki-67 lower than 90% [39,40]; using these specific parameters for selecting cases for FISH testing seems to be inappropriate. These findings were also reflected in the current study, with less than 75% of DH cases being of GCB origin, ~50% with high proliferative index and ~15% not displaying high c-MYC expression. Therefore, new screening methods are direly needed.

In light of this unmet clinical need and considering that AI-based solutions offer accessible tools that can provide a biomarker status within minutes, we assessed whether the AI DHL-classifier could provide an alternative screening tool for aggressive DH B-cell lymphoma cases. Although our cohort size was small, our preliminary data demonstrated that the classifier effectively detected DHL cases with a predictive value of 92%, capturing all FISH-positive cases. Confirmation in a larger cohort, representing a more diverse group of patients, is required. However, our results, if confirmed, suggest that the DHL AI-based classifier may serve as a useful screening tool in places where FISH analysis is limited.

Our study has several limitations, mainly attributed to its retrospective nature and the small cohort size. During the study period, FISH was not routinely performed in our institution, but was reserved for patients with highly aggressive disease and/or a high Ki-67 proliferative index, introducing a selection bias of cases that were more likely to be positive. Moreover, the number of patients included in our study was small and all the samples were attained from a single center. All these factors, together with the known impact of scanning devices, specimen processing and staining protocols on histological slide images [41], emphasize the need for validating our results in a large prospective cohort, representing a more diverse group of patients, in order to assess the robustness and generalizability of our DHL-classifier. Additionally, NGS was not performed in any of the cases; thus, it is impossible to conclusively determine whether our false positive cases represent DHL cases with cryptic translocations. Therefore, evaluating samples with NGS in addition to the traditional FISH in follow-up studies can be of value for a more detailed investigation and characterization of the cohort, particularly in the case of false positives.

Moreover, the definition of HGBL-DH has been recently changed, referring now to patients with *MYC/BCL2* rearrangement only [42]. Therefore, a new algorithm, differentiating between *MYC/BCL2* and *MYC/BCL6*-rearraged cases, is currently warranted.

Lastly, while our algorithms’ lack of interpretability poses challenges in model refinement and obtaining insights that can drive advances in the molecular pathology realm, we are optimistic that future advancements in explainable AI algorithms will facilitate substantial improvements in this field.

## 5. Conclusions

We presented here a proof of concept for the potential use of a DL algorithmic tool for the identification of DH/TH events, promoting the performance of FISH in “DH/TH-suspected” cases only. While further investigation, development and implementation of the proposed tool are required, it would be of great interest to further investigate the impact of integrating such a tool within the clinical workflow. Moreover, it would be of great interest to explore if such a tool could also be used for other lymphomas, for example, the identification of *MYC* translocations in Burkitt lymphoma, and to establish whether such AI solutions can improve hematological cancer patients’ care.

## Figures and Tables

**Table 1:** Patient characteristics.

	Entire Cohort (*n* = 57)	Training Set (*n* = 32)	Validation Set (*n* = 25)
Male, *n* (%)	32 (56.1)	18 (56.3)	14 (56)
Age (years), median (range)	62 (8–84)	66.5 (8–84)	60 (17–77)
Tested tissue, *n* (%)			
Lymph node	22 (38.6)	16 (50.0)	6 (24.0)
Extra nodal	35 (61.4)	16 (50.0)	19 (76.0)
Procedure, *n* (%)			
Needle biopsy	43 (75.4)	24 (75.0)	19 (76.0)
Excisional	14 (24.6)	8 (25.0)	6 (24.0)
ECOG PS, *n* (%) *			
0/1	38 (82.6)	24 (82.8)	14 (82.4)
≥2	8 (17.4)	5 (17.2)	3 (17.6)
Disease stage *			
I/II	13 (24.5)	7 (23.3)	6 (26.1)
III/IV	40 (75.5)	23 (76.7)	17 (73.9)
LDH level *			
Normal	14 (28.0)	10 (32.3)	4 (21.1)
Increased	36 (72.0)	21 (67.7)	15 (78.9)
COO, *n* (%) #*			
GCB	27 (52.9)	13 (46.4)	14 (60.9)
IHC			
Ki67 *			
Median % (range)	80 (10–100)	80 (10–100)	85 (40–100)
Ki67 ≥ 90%	23 (41.8)	12 (37.5)	11 (47.8)
c-MYC expression *			
Positive/borderline positive	33 (61.1)	20 (64.5)	13 (56.5)
Negative	21 (38.9)	11 (35.5)	10 (43.5)
BCL2 expression *			
Positive/borderline positive	33 (62.3)	21 (67.7)	12 (54.5)
Negative	20 (37.7)	10 (32.3)	10 (45.5)
BCL6 expression *			
Positive/borderline positive	49 (89.1)	29 (90.6)	20 (87.0)
Negative	6 (10.9)	3 (9.4)	3 (13.0)
FISH			
DHL/THL, *n* (%)	15 (26.3)	5 (15.6)	10 (40)

ECOG PS—Eastern Cooperative Oncology Group performance status; COO—cell of origin; LDH—lactic dehydrogenase; GCB—germinal center B-cell; IHC—immunohistochemistry; FISH—fluorescence in situ hybridization; DHL/THL — double/triple-hit lymphoma; % are depicted from the total *n* with information per criteria. # GCB—based on HANS immunohistochemical criteria; * missing data: ECOG PS (*n* = 11), disease stage (*n* = 4), LDH (*n* = 7), COO (not determined/equivocal; *n* = 6), Ki-67% (*n* = 2), c-MYC (*n* = 3), BCL2 (*n* = 4) and BCL6 (*n* = 2).

## Data Availability

The datasets generated during the current study are not publicly available and are available upon reasonable request.

## Supplementary Materials

The following supporting information can be downloaded at: https://www.mdpi.com/article/10.3390/cancers15215205/s1, Table S1: Predictive values of conventional criteria *vs*. the AI DHL-classifier for correctly deciding whether to perform FISH testing.
