# Supplementary material for: Image-Based Deep Learning Detection of High-Grade B-Cell Lymphomas Directly from Hematoxylin and Eosin Images
Source: Cancers (Basel). 2023 Oct 29;15(21):5205. doi: 10.3390/cancers15215205 (PMC10650414; doi:10.3390/cancers15215205)
Supplement: Supplementary file 1 [file cancers-15-05205-s001.zip › cancers-2565451-supplementary.pdf]

Supplementary Table S1:

Predictive values of conventional criteria *vs.* the AI DHL-classifier for correctly deciding whether to perform FISH testing.

Method	Total	# N	# P	# TN	# TP	# FN	# FP	sensitivity	specificity	accuracy
**DHL-classifier**	25	15	10	13	10	0	2	100%	86.7%	92%
**Ki-67 ≥90%**	23	14	9	8	5	4	6	55.6%	57.1%	56.5%
**c-MYC expression**	23	14	9	9	8	1	5	88.9%	64.3%	73.9%
**GCB**	23	13	10	7	8	2	6	80%	53.9%	65.2%
**Ki-67 or c-MYC or GCB**	23	14	9	4	9	0	10	100%	28.6%	56.5%

N-negative, P-positive, TN-true negative, TP-true positive, FN-false-negative, FP-false positive, GCB- germinal center B cell
